# Constant Illumination Boosts Rice Immunity via the Nicotinamide Mononucleotide Signaling Pathway: A Mechanistic Exploration

**DOI:** 10.1186/s12284-026-00892-x

**Published:** 2026-02-20

**Authors:** Mengyan Sun, Xinqing Wu, Kunying Ding, Lin Song, Mengying Zhou, Xiaoyan Su, Zhi Ye, Minghao Tang, Tao Lu, Haifeng Qian, Zhengwei Fu, Yinhua Ni

**Affiliations:** 1 College of Biotechnology and Bioengineering, Zhejiang University of Technology, No.6 District, Zhaohui, Hangzhou, 310032 Zhejiang China; 2 College of Environment, Zhejiang University of Technology, Hangzhou, 310032 China

**Keywords:** Circadian clock, Plant immunity, Rice blast, Constant light, NMN

## Abstract

Light serves as a key input signal for the plant circadian clock, and together they coordinately regulate plant growth and development. However, the nature of their relationship regarding rice immunity remains unclear. The present study aimed to clarify the mechanism by which light modulates rice immunity through the circadian clock. The results indicated that constant light exposure significantly alleviated rice blast symptoms, while constant dark exposure exacerbated them. Transcriptome analysis demonstrated that constant light altered the phases of core circadian clock genes and activated circadian-controlled pathways, including the nicotinamide mononucleotide (NMN)-associated energy metabolism pathway. Metabolomics analysis and experimental validation confirmed that constant light exposure significantly increased the level of NMN, nicotinamide adenine dinucleotide (NAD^+^), and nicotinamide adenine dinucleotide phosphate (NADP⁺). Notably, exogenous NMN application effectively mitigated rice blast symptoms. Our study provided critical insights into the interplay among light, the circadian clock, and plant immunity, offering potential applications in agricultural production.

**Supplementary Information:**

The online version contains supplementary material available at 10.1186/s12284-026-00892-x.

## Introduction

The plant circadian clock is a sophisticated endogenous timing network that provides plants with an adaptive advantage for growth and development by coordinating cellular physiology, metabolic activities, and various developmental processes in a temporal dimension (Xu et al. 2022). The plant circadian clock system is mainly composed of input pathways, core oscillators, and output pathways (Hsu and Harmer 2014; Nohales and Kay 2016). Environmental factors such as light, temperature, and humidity serve as primary input pathways that influence the core oscillator of the circadian clock (Xu et al. 2022). Among these environmental factors, light is a pivotal regulator that influences all primary developmental stages of plants (Wu et al. 2024). The core oscillator is a complex molecular regulatory network consisting of multiple interconnected transcription-translation feedback loops (TTFLs) (Nakamichi et al. 2022). TTFLs are primarily composed of the transcriptional repressors *circadian clock-associated 1* (*CCA1*), *late elongated hypocotyl* (*LHY*), and *timing of cab expression 1* (*TOC1/PRR1*) (Dixon et al. 2011; Farré and Liu 2013). However, few studies have examined how the core oscillator of the plant circadian clock changes after altering the plant’s input pathway. Previous studies have shown that photoreceptors mediate the input of light signals to the circadian clock, with light rapidly inducing the expression of multiple core clock genes (Nohales and Kay 2016). For instance, even a brief 90-second red light pulse can trigger expression of the central circadian gene *CCA1* (Wang and Tobin 1998). Furthermore, exposing etiolated seedlings to different wavelengths of light for approximately 2 h can rapidly induce the expression of multiple core circadian clock genes, including *CCA1*, *LHY*, *pseudo-response regulator 9* (*PRR9*), *night light-inducible and clock-regulated 1 (LNK1*), *night light-inducible and clock-regulated 3* (*LNK3*), *early flowering 4* (*ELF4*), and *TOC1* (Makino et al. 2001; Tepperman et al. 2001). These genes play pivotal roles in the plant circadian regulatory network, and their expression changes directly reshape the oscillatory dynamics of the circadian clock (Makino et al. 2001). Different photoperiods can alter the expression phase of circadian clock genes *PRRs* and prolong their expression duration (Li et al. 2020). This rapid light-mediated modulation of key circadian components carries profound biological significance, enabling plants to adjust their physiological rhythms in response to ambient light conditions, thereby enhancing environmental adaptation (Michael et al. 2008). However, whether long-term alterations in light duration affect the plant circadian clock and thereby more significantly modulate its physiological output pathways remains unclear.

The output pathways of the plant circadian clock are extensive, regulating nearly all physiological and biochemical processes in plants (Adams and Carré 2011). Among these, plant immunity is one of the key output pathways regulated by the circadian clock (Harmer 2009; Grundy et al. 2015). The regulation of plant immune responses by the circadian clock is reflected in multiple aspects, including the expression of resistance genes, hormone synthesis and signaling, reactive oxygen species (ROS) homeostasis, and ion balance (Sharma and Bhatt 2015). Plants employ a two-layered defense system to protect themselves against pathogens. The first layer involves pattern recognition receptors (PRRs) on the cell surface (Couto and Zipfel 2016), which detect conserved microbial molecules known as pathogen-associated molecular patterns (PAMPs) (Singh et al. 2022). This recognition activates pattern-triggered immunity (PTI) (Yu et al. 2024), serving as the plant’s first line of defense against infection (Yuan et al. 2021a, b). The second layer of plant immunity is effector-triggered immunity (ETI), which represents a more precise immune response. ETI specifically recognizes effector proteins secreted by pathogens into plant cells, thereby activating a series of stronger immune reactions (Yuan et al. 2021a, b; Chang et al. 2022). Furthermore, the plant circadian clock mediates the synthesis and signaling of immune-related hormones. Plant immune receptors frequently activate multiple hormonal pathways, including salicylic acid (SA) and jasmonic acid (JA) signaling cascades(Li et al. 2018; Zhang et al. 2019). These phytohormone pathways play crucial roles in defending against diverse pathogenic microorganisms (Bürger and Chory 2019). Beyond plant immune hormones, it remains an important question whether the circadian clock also regulates other metabolites to modulate immune responses.

Rice blast, a devastating fungal disease caused by (*Magnaporthe oryzae*, *M. oryzae*) (Liu et al. 2013), occurs across all rice-growing regions in China. In severely affected fields, it can lead to complete crop failure (Chakraborty et al. 2021). Recognized as one of the three major rice diseases alongside rice sheath blight and bacterial leaf blight, rice blast poses a significant threat to rice production in China (Asif et al. 2022). Rice blast management should not rely on a single strategy but instead establish a diversified, systematic control framework. Our previous study demonstrated that the rice circadian clock participates in the response to rice blast infection by regulating rice metabolism and the calcium signaling pathway (Sun et al. 2025). The circadian clock can precisely regulate the expression of rice immunity-related genes, enabling them to function most effectively at specific times and thus optimizing the rice immune response (Sun et al. 2025). Under overcast and rainy conditions with insufficient sunlight, rice blast disease is prone to large-scale outbreaks. Previous studies have shown that light can induce phosphorylation of a chloroplast protein, the light-harvesting complex II protein (LHCB5), thereby activating immune responses to resist rice blast disease (Liu et al. 2019, 2025). Light modulates plant immunity through multiple pathways (Ballaré 2014). It activates resistance-gene-mediated hypersensitive responses by promoting hydrogen peroxide (H₂O₂) accumulation, enhancing ascorbate peroxidase and catalase activities, and regulating hormone signaling (Chandra-Shekara et al. 2006). These responses confer resistance against bacteria, fungi, and herbivores (Chandra-Shekara et al. 2006). Research has revealed that constant light exposure can enhance *Arabidopsis thaliana*’s resistance to *Pseudomonas syringae pv. tomato* (*Pst*) by inducing increased SA production, thereby promoting plant immunity (Lajeunesse et al. 2023).

Light regulates the plant circadian clock, enabling it to align with fluctuating environmental conditions. However, the precise mechanisms by which light modulation alters circadian rhythms to coordinate immune responses during rice blast infection remain unknown. Therefore, this study aims to identify key pathways and metabolites involved in circadian-controlled immune response during rice blast infection under different light regimes using transcriptomic and metabolomic approaches. How the circadian clock coordinates rice immune responses under altered light conditions will be thoroughly investigated, and the results may provide a theoretical foundation for understanding the complex relationships among light, the circadian clock, and rice immunity. Importantly, optimizing light management to enhance disease resistance and developing novel green control strategies based on circadian regulation may also reduce pesticide usage and offer new insights for sustainable agricultural development.

## Materials and Methods

### Plant Material and Fungi Strains

The indica rice variety 9311 was cultivated in a greenhouse maintained at 28℃ under a 12-h light/12-h dark photoperiod (light on at 8:00 am, and off at 20:00 pm). The *M. oryzae* strain Guy11 was grown on solid complete media (CM) for 7 to 10 days at 28℃. Conidiation was stimulated by placing the culture under a black lamp for 7 to 10 days on a straw-decomposition culture (SDC) medium at 28℃, as described previously (Sun et al. 2025).

### Pathogen Infection

The experiment used a two-week-old indica rice variety, 9311, at the two-leaf-one-heart stage. The rice was placed in a 24℃ incubator with a 12 h/12 h light/dark cycle and 90% relative humidity to acclimate for one day. On the second day, a conidial suspension at approximately 5 × 10⁵ spores/mL (0.05% Tween) was prepared. The rice was infected with the suspension at 20:00 pm (T0), and the suspension was evenly sprayed onto the rice leaves using a spray bottle. Under identical conditions, control group samples were treated with an equivalent volume of sterile water containing 0.05% Tween via spray application. The plants were then returned to the incubator. After infection, the incubator was kept in continuous darkness for 24 h, and then the plants were subjected to normal light (DL), constant light (LL), and constant dark (DD) conditions at 24–48 h post-infection (T24-48). Samples from the control and infected groups were collected every 6 h from 24 h post-infection until 96 h. The samples collected at 48 h post-infection were used for transcriptome and metabolome sequencing and stored at −80℃ for later use. Detailed photographs were taken to record the blast lesions after 5 days of infection.

### Treatment with Nicotinamide Mononucleotide

Before treatment, the rice was incubated at 24℃ and 80% relative humidity under a 12 h/12 h light/dark photoperiod to acclimate. Both in vivo and in vitro assays were conducted separately. For the in vivo experiments, 100 µM nicotinamide mononucleotide (NMN) was sprayed onto the rice plants at 16:00 pm (4 h prior to rice blast infection), followed by *M. oryzae* infection at 20:00 pm with 90% relative humidity. Subsequently, rice leaf samples from each in vivo group were collected at 6-h intervals for 48 h after rice blast infection. In contrast, the in vitro experiments employed a droplet application method on leaf segments, as described previously (Yan et al. 2023). Briefly, sterile filter paper moistened with sterile water was placed in a culture dish. Rice seedlings at the two-leaf stage were selected, and leaf segments approximately 5 cm in length were cut from the same position of each seedling, while placing the leaves on the filter paper. Four hours before rice blast infection (at 16:00 pm), 10 µL droplets of NMN at concentrations of 50 and 100 µM were separately applied to the leaf segments at intervals of 1.5 cm. At 20:00 pm, the NMN solution was removed by blotting with filter paper, followed by the application of an M. oryzae spore suspension at a concentration of 5 × 10⁵ spores/mL. For the control group, an equivalent volume of sterile water was applied instead. Approximately three droplets were applied per leaf segment.

### Determination of H₂O₂ in Rice Leaves

H₂O₂ in rice leaves was detected using 3,3’-diaminobenzidine (DAB, 0430, Gentihold, Beijing, China) staining. DAB solution was prepared by dissolving 1 mg of DAB in 1 mL of water and adjusting the pH to 5.8. Rice leaf samples were rinsed twice with sterile water and then immersed in DAB solution for 24 h in the dark. Subsequently, the samples were decolorized in a solution of 100% ethanol: acetic acid (3:1) for 24 h until the leaves were completely decolorized. The decolorized leaves were then mounted on slides and observed under an inverted microscope for signs of H₂O₂ accumulation. In the presence of peroxidase, DAB is oxidized by H₂O₂ to generate insoluble dark brown precipitates. Accordingly, a larger area of dark brown staining corresponds to a higher H₂O₂ content. For H₂O₂ quantification, the micrographs were first converted to grayscale in ImageJ, followed by threshold-based segmentation to distinguish the background from regions with staining intensity above the predefined threshold. H₂O₂ deposition was quantified as the percentage of the above-threshold stained area relative to the total leaf area.

### Rice Leaf Callose Analysis

Callose deposition was detected using aniline blue (S19056, Source Leaf, Shanghai, China). Leaves to be tested were immersed in a decolorizing solution composed of phenol: glycerol: lactic acid: water:100% ethanol (1:1:1:1:2). Once decolorization was complete, the leaves were gently rinsed with sterile water and then placed in 3–5 mL of 150 mM aniline blue solution in the dark for 24 h. The stained leaves were then mounted in glycerol, rinsed gently with water, and observed and photographed under UV excitation using a fluorescence microscope. For callose deposition analysis, the bright fluorescent foci in the images represent callose deposits. Callose quantification was performed subsequent to grayscale conversion by ImageJ: the software converted color micrographs to grayscale, and a threshold was set to categorize pixels into two cohorts. Pixels with intensity exceeding the threshold were defined as callose deposition sites, while those below the threshold were classified as background. The software then counted the number of callose-positive pixels and converted the pixel count to absolute area units.

### **NAD**^**+**^**and NADP**^**+**^**Extraction and Quantification**

Rice leaves were homogenized on ice at a 1:10 (w/v) ratio in acidic extraction buffer, incubated in a 95 °C water bath for 5 min, and immediately cooled. After centrifugation (10,000 g, 10 min, 4 °C), 500 µL of the supernatant was neutralized with 500 µL of alkaline extraction buffer, recentrifuged under identical conditions, and the final supernatant was stored on ice. NAD^+^ (m1092586) and NADP^+^ (m1076443) were detected using the corresponding kits (Enzyme-linked Biotechnology Co., Shanghai, China) according to the manufacturer’s instructions. The acidic and alkaline extraction solutions used in this study were supplied as finished products by Enzyme-Linked BioTech Co., Ltd, along with the ELISA kits.

### Extraction and Determination of JA and SA

The weighted leaves were ground into powder using liquid nitrogen. For JA determination, an appropriate amount of pre-cooled PBS was mixed with the sample at a weight-to-volume ratio of 1:9. JA was detected using an ELISA kit (Enzyme-linked Biotechnology Co., CB10072, Shanghai, China) according to the manufacturer’s instructions. For the extraction and determination of SA, leaves were mixed with the solution of water: isopropanol: hydrochloric acid (2:1:0.0002) at a proportion of 1 g/100 mL. The mixtures were placed on a shaker at 4 °C at 100 r/min for 30 min. Then, 1 mL of dichloromethane was added, and the mixture was shaken for another 30 min. The mixture was centrifuged at 13,000 g for 5 min, and the lower layer was collected. The solution was dried using a nitrogen blower, then dissolved in 150 µL of methanol and filtered through a 0.22 μm filter membrane for subsequent testing. SA levels were analyzed using a high-performance liquid chromatography (HPLC) system (Thermo Fisher, U3000, USA).

### RNA Extraction and qRT-PCR Analysis

The fresh leaves were collected from the control and rice blast-infected groups and ground to a powder in liquid nitrogen for RNA extraction. Total RNA from rice leaves was extracted using Trizol reagent (Vazyme, Nanjing, China) as described previously (Hu et al. 2026; Zheng et al. 2026), and the RNA concentration was assessed using a Nano-3000 instrument (Aosens, Hangzhou, China). The cDNA was synthesized using a high-capacity cDNA reverse transcriptase kit (Vazyme, Nanjing, China), and qPCR was performed using SYBR Green Real-time PCR Master Mix (Vazyme, Nanjing, China) on a CFX Connect Real-Time PCR System (Bio-Rad, USA). The ubiquitin transcript was used as a housekeeping gene for data standardization. The primer sequences are listed in Table S1.

### Transcriptome Analysis

Total RNA was extracted from rice leaves using TRIzol^®^ reagent (Invitrogen, USA). The RNA sequencing was conducted at Shanghai Biozeron Biotechnology Co., Ltd (Shanghai, China). Gene Ontology (GO) enrichment analysis was performed using GOatools (https://github.com/tanghaibao/GOatools), and Kyoto Encyclopedia of Genes and Genomes (KEGG) pathway enrichment analysis was conducted using KOBAS (http://kobas.cbi.pku.edu.cn/kobas3/?t=1). The expression levels of genes were measured using Fragments Per Kilobase of Exon Model Per Million Mapped Reads (FPKM) values. The criteria for screening differentially expressed genes were |logFC| ≥ 1 and FDR ≤ 0.05.

### Metabolomics Analysis

Untargeted metabolomics analysis using UHPLC-MS/MS was performed at Shanghai Biozeron Biotechnology Co., Ltd (Shanghai, China). Data were analyzed using the Variable Importance in Projection (VIP) values from the Orthogonal Partial Least Squares Discriminant Analysis (OPLS-DA) model, in conjunction with the p-values from independent samples t-tests to identify differentially expressed metabolites. The screening criteria for differential metabolites were set as: |log2FC|≥1, OPLS-DA VIP ≥ 1, and p-value ≤ 0.05. Metabolite classification was annotated using the Human Metabolome Database (HMDB, https://hmdb.ca/metabolites).

### Statistical Analysis

All data are given as the mean ± SEM. Comparisons between different groups were analyzed by one-way analysis of variance (ANOVA) followed by the Tukey-Kramer test. Significance levels were set at ^*/#^*p* < 0.05, ^**/##^*p* < 0.01. Rhythm analysis was performed using MetaCycle in R. The R package incorporates ARSER, JTK_CYCLE, and Lomb-Scargle to conveniently evaluate periodicity in time-series data, including calculating rhythmic *p*-values, amplitudes, phases, and periods (Wu et al. 2016).

## Results

### Different Light Conditions Regulated Rice Immunity

**Fig. 1:**
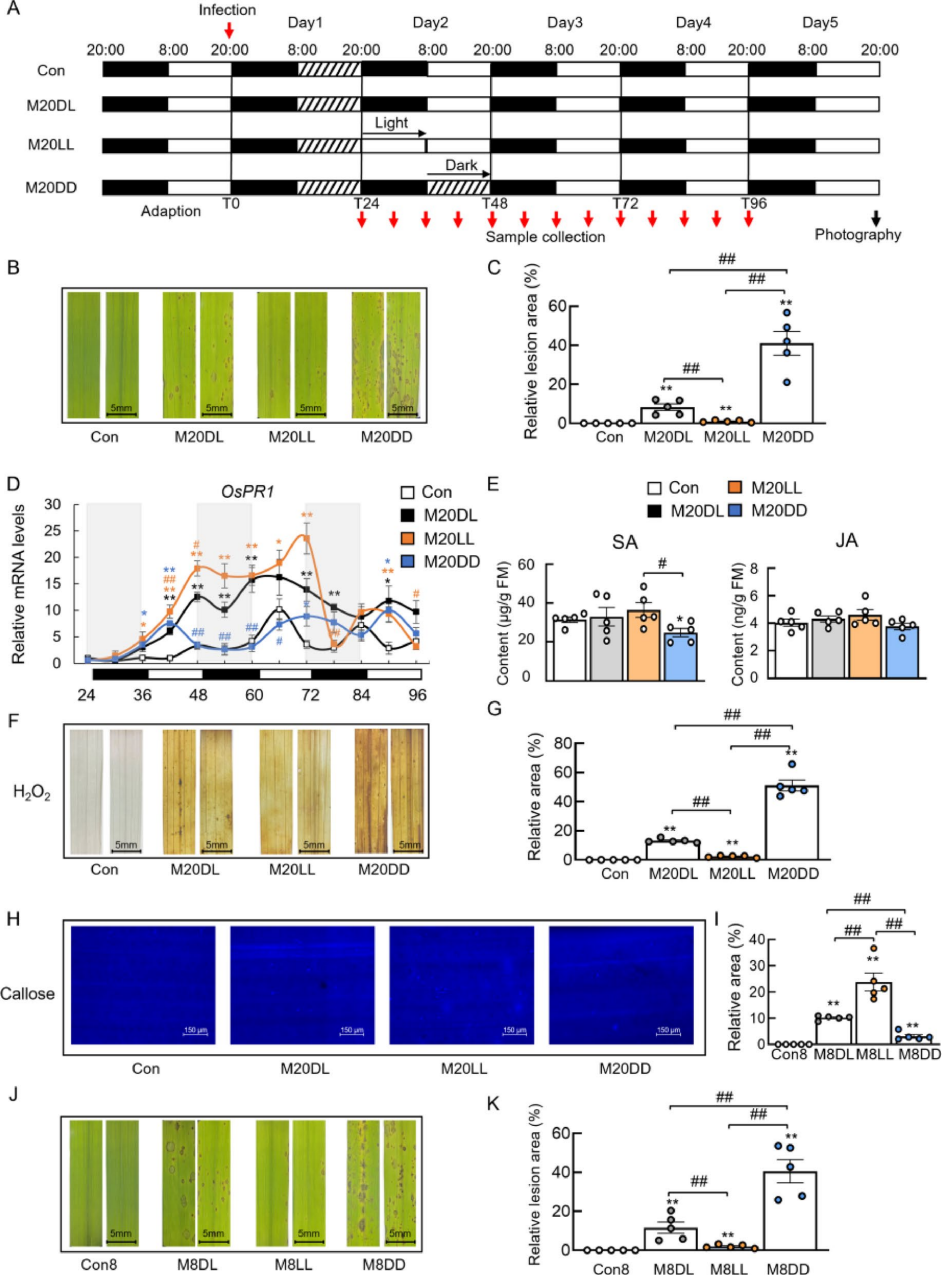
Constant light exposure alleviated rice blast symptoms. **A** Experimental design for rice blast infection at 20:00 pm, the shaded area (with slanted lines) in the diagram denotes the region of artificial darkness. **B** Leaf phenotypes after 5 days of rice blast infection at 20:00 pm, the scale bar represents 5 mm. **C** Quantification of lesion areas on rice leaves. **D** RT-qPCR analysis of the rhythmic expression of *OsPR1* under different light regimes. **E** Quantification of the plant immune hormones SA and JA. **F** H₂O₂ accumulation, the scale bar represents 5 mm. **G** H₂O₂ quantification. **H** Callose deposition, the scale bar represents 150 μm. (I) Callose deposition quantification. **J** Leaf phenotypes 5 days of rice blast infection at 8:00 am, the scale bar represents 5 mm. **K** Quantification of lesion areas on rice leaves. Data are expressed as means ± SEM, *n* = 5 for each group for qPCR and phytohormone analysis, *n* = 5 for lesion quantification, *n* = 5 for H₂O₂ quantification, *n* = 5 for callose deposition quantification, ^*^p < 0.05, ^**^p < 0.01 vs. Con, ^#^*p* < 0.05, ^##^*p* < 0.01 vs. M20DL or as indicated

To investigate the regulatory effects of different light regimes on rice immunity, four groups were established: the normal dark/light control group (Con20DL/Con), the normal dark/light infection group (M20DL), the constant light infection group (M20LL), and the constant dark infection group (M20DD). Rice was infected at 20:00 pm, and the leaf phenotypes of the rice were observed on the 5th day (Fig. 1A). It was found that the symptoms of rice were alleviated in the M20LL group, while the M20DD group showed a certain degree of worsening of blast symptoms (Fig. 1B, C). Subsequently, RT-qPCR was used to verify the rhythmic expression of the disease-resistant gene *Oryza sativa* pathogenesis-related 1 (*OsPR1*). The expression of the *OsPR1* gene in the M20LL group began to increase 6 h after constant light exposure, and overall, the expression level of *OsPR1* was higher under M20LL conditions than under M20DL and M20DD conditions (Fig. 1D). However, this effect persisted only until 72 h post-treatment. During the subsequent period from 72 to 96 h, the expression level of *OsPR1* declined (Fig. 1D). Next, the levels of the plant immune hormones, mainly the SA and JA, were measured at 48 h post-rice blast infection. It was found that the SA content was highest under M20LL conditions and lowest under M20DD conditions. However, there was no significant change in JA content under different light conditions (Fig. 1E). The accumulation of H_2_O_2_ and callose under different light conditions was also measured. The M20DD group produced the most H_2_O_2_, while the M20LL group produced the least, which might be due to the highest degree of leaf damage in the M20DD group (Fig. 1F, G). And the M20LL group displayed more callose deposition, while the M20DD group showed the least callose accumulation (Fig. 1H, I).

To further confirm the immunity-boosting effect under constant light exposure, a similar experiment with rice blast infection at 8:00 am was performed (Fig. S1A). Similarly, M8LL alleviated disease symptoms, whereas M8DD exacerbated rice blast lesions (Fig. 1J, K). RT-qPCR analysis of collected leaves revealed that OsPR1 expression was highest in M8LL compared with M8DL and M8DD at 48 h post-infection (Fig. S1B). SA and JA contents followed the same trends observed under 20:00 pm infection (Fig. S1C). Likewise, H₂O₂ accumulation and callose deposition at 48 h mirrored the patterns found at 20:00 infection (Fig. S1D–G). Collectively, the immune response of rice to blast infection was enhanced under a constant light condition, whereas the symptoms of rice blast disease were exacerbated under a constant dark condition.

### Constant Light Exposure Modulated Photosynthesis and Metabolism Through the Circadian Clock

**Fig. 2:**
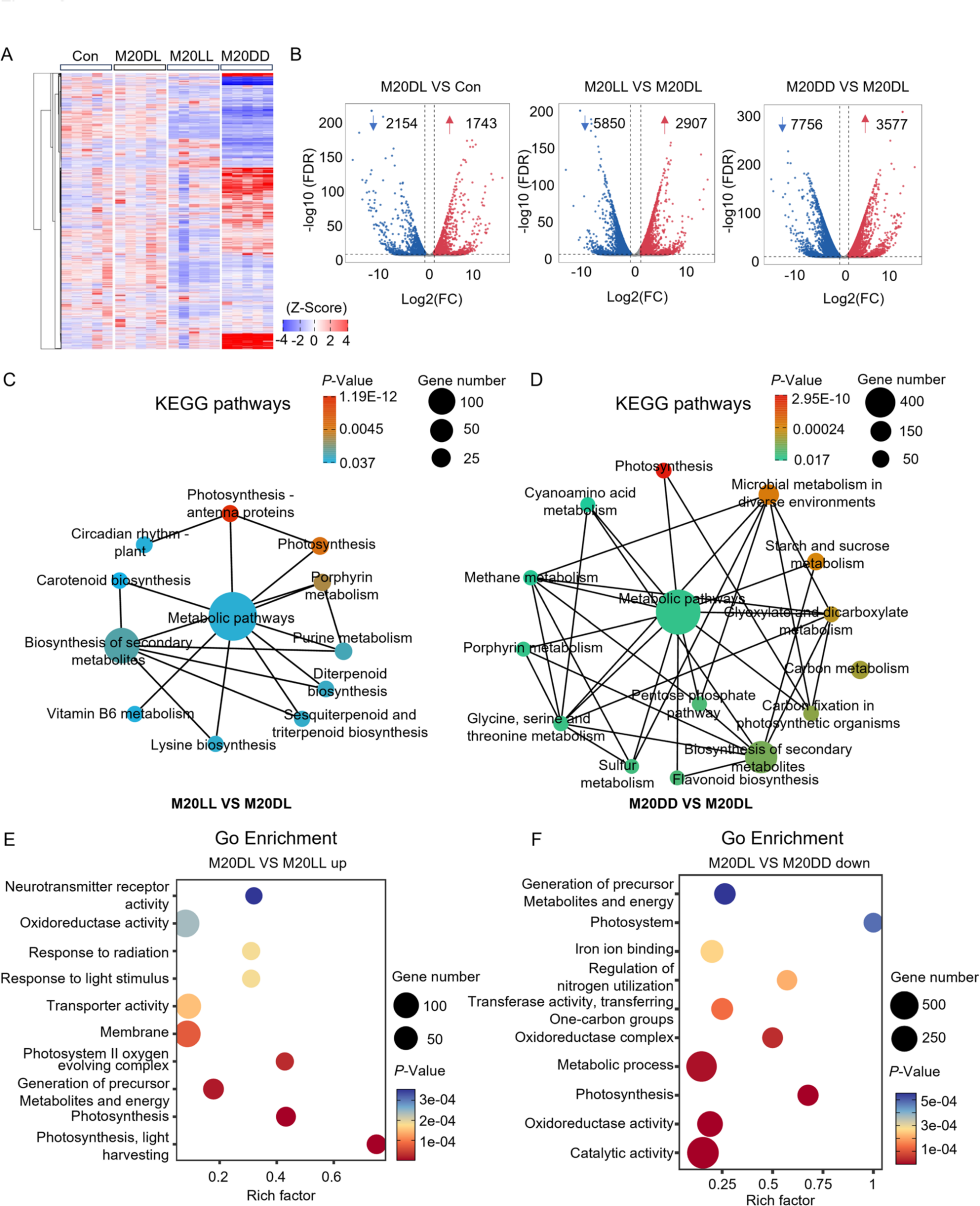
Constant light modulated photosynthesis and metabolism via the circadian clock. **A** Heatmap showing transcriptional changes under different light regimes. **B** DEGs among the groups under different light regimes. **C** KEGG pathway enrichment of DEGs in M20LL vs. M20DL. **D** KEGG pathway enrichment of DEGs in M20DD vs. M20DL. **E** GO enrichment of upregulated DEGs in M20LL vs. M20DL. **F** GO enrichment of down-regulated DEGs in M20DD vs. M20DL. DEGs were filtered with |log₂FC| ≥ 1 and FDR ≤ 0.05

Subsequently, RNA-seq was performed on samples collected 48 h after infection with *M. oryzae* to dissect how light conditions influence gene expression systematically. Hierarchical clustering heatmaps revealed markedly distinct transcriptional patterns among the four groups (Fig. 2A). A total of 1743 genes were significantly upregulated and 2154 genes were significantly downregulated in the M20DL group, compared the the control group (Fig. 2B). The significant differentially expressed genes (DEGs) were 2907 and 3577 upregulated, and 5850 and 7756 downregulated in the M20LL and M20DD groups when compared to the M20DL group, respectively (Fig. 2B). These results indicated that altering the light environment exerted broad transcriptional regulation and that gene expression landscapes diverged sharply across different light conditions. Next, KEGG pathway annotation and GO enrichment analyses were conducted. KEGG profiling of DEGs in M20DL compared to the control group showed that blast infection primarily perturbed diterpenoid biosynthesis and robustly activated the MAPK immune-signaling pathway, as well as phenylpropanoid metabolism, plant hormone signal transduction, and glutathione metabolism, which are well-established pathways in plant immunity (Fig. S2A). When compared to the M20DL group, constant light regimen markedly rewired the plant circadian rhythm pathway, photosynthetic antenna proteins, photosynthesis, porphyrin metabolism, purine metabolism, and terpenoid biosynthesis (Fig. 2C). In contrast, constant dark enriched DEGs in photosynthesis, microbial metabolism in diverse environments, starch and glycogen metabolism, carbon metabolism, and carbon fixation in photosynthetic organisms (Fig. 2D). Collectively, these results showed that light regimes profoundly reshaped energy supply and metabolic homeostasis in rice following blast infection.

On the other hand, the top 10 GO enrichment revealed that upregulated genes in the M20DL group were enriched in catalytic activity, ion binding, oxidoreductase activity, and monooxygenase activity (Fig. S2B). In addition, the genes upregulated in the M20LL group compared to the M20DL group revealed that constant light exposure activated processes such as photosynthesis, light harvesting, generation of precursor metabolites and energy, the oxygen-evolving complex of photosystem II, and plasma membrane components (Fig. 2E). In contrast, the genes downregulated in the M20DD group indicated that constant dark regimen attenuated the pathways related to photosynthesis, generation of precursor metabolites and energy, catalytic activity, and oxidoreductase activity (Fig. 2F). Taken together, the coupling of circadian rhythm with photosynthesis indicated that light regimen alteration may regulate downstream metabolic cascades via clock-mediated control of photosynthetic gene expression.

### Constant Light Altered the Phase and Amplitude of Circadian Clock Gene

**Fig. 3:**
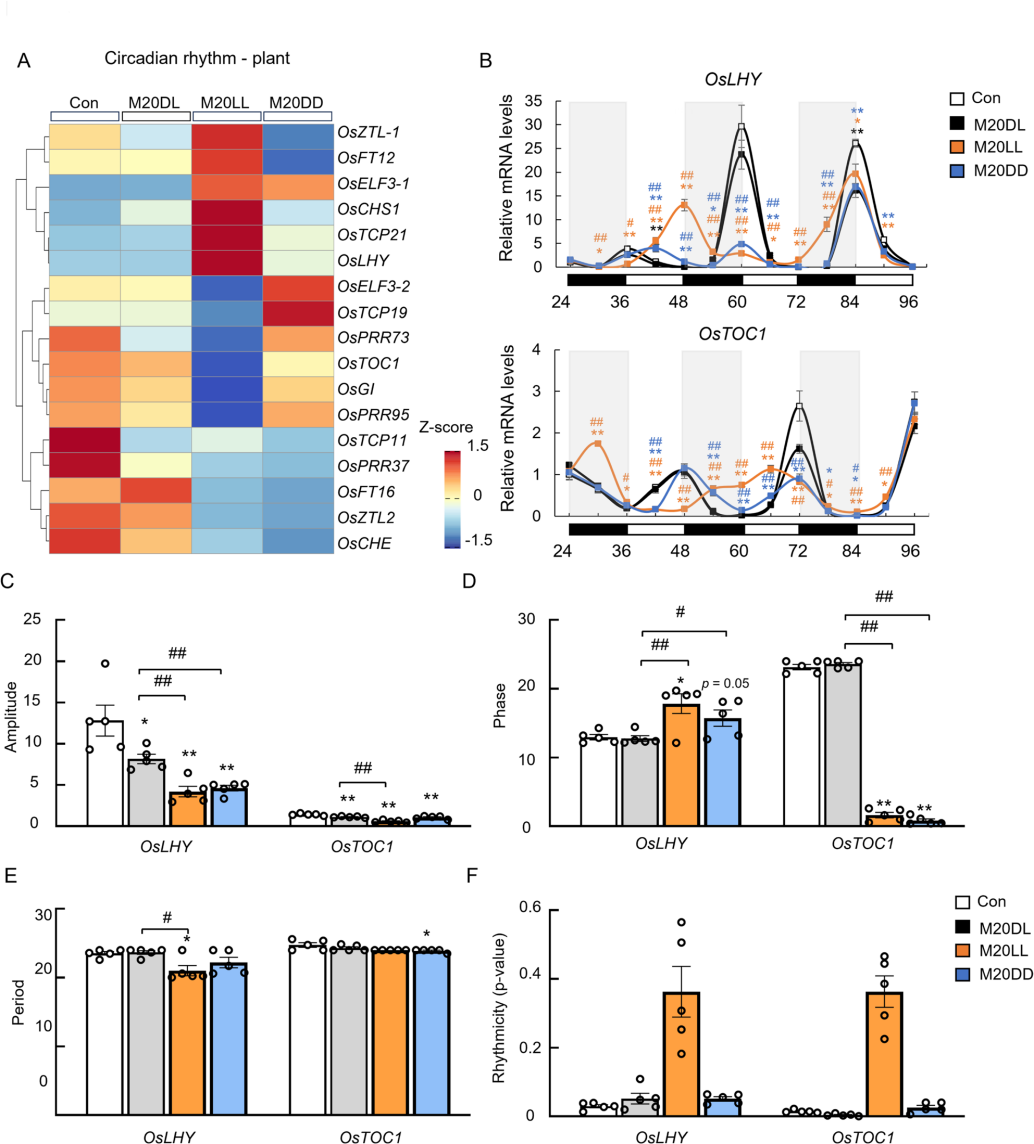
Constant light altered the rhythm of circadian clock genes. **A** Heatmap of core circadian clock genes. **B** RT-qPCR validation of the rhythmic expression of core clock genes *OsLHY* and *OsTOC1* at 24–96 h after rice blast infection. **C**–**F** Amplitude, phase, period, and rhythmicity analyses of *OsLHY* and *OsTOC1*. Data are expressed as means ± SEM, *n* = 5 for each group for transcriptome and qPCR analysis, ^*^p < 0.05, ^**^p < 0.01 vs. Con, ^#^*p* < 0.05, ^##^*p* < 0.01 vs. M20DL

To further clarify the impact of light alteration on the circadian clock, the core circadian clock genes of rice were visualized by a heatmap. The results showed that expression of these genes was strongly affected under both constant light and dark regimes (Fig. 3A). Next, RT-qPCR analysis of the rhythmic expression of the central clock genes, mainly *Oryza sativa* late elongated hypocotyl (*OsLHY*) and *Oryza sativa* timing of CAB expression 1 (*OsTOC1*), over a 24–96 h time-course after rice blast infection was performed. Different photoperiods up- or downregulated the expression of *OsLHY* and *OsTOC1* at distinct time points. Overall, the amplitude of *OsLHY* was significantly reduced under both constant light and constant dark conditions, whereas the amplitude of *OsTOC1* was reduced considerably only under constant light (Fig. 3B–C). Both constant light and constant dark delayed the phase of *OsLHY*, while the phase of *OsTOC1* was advanced (Fig. 3D). In contrast, different light conditions had a minor effect on the period of the core circadian genes (Fig. 3E). Notably, constant light led to a pronounced loss of rhythmicity (Fig. 3F). Collectively, these results revealed that constant light regimen exerted a stronger effect on the circadian clock than constant dark, yet the impact of both regimes peaked between 24 and 72 h after infection and declined thereafter.

### Constant Light Activated the Production of Energy-Related Metabolites

**Fig. 4:**
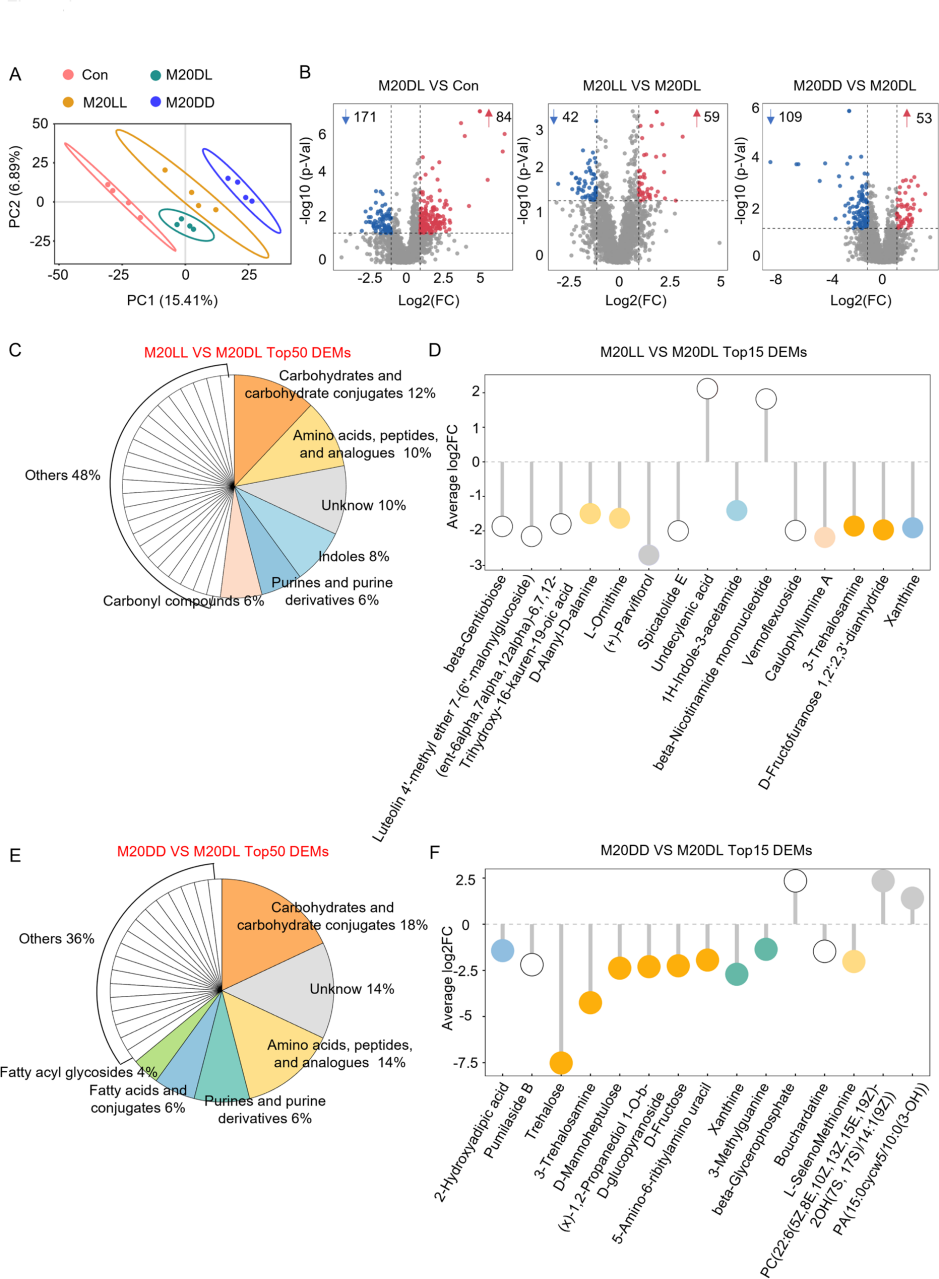
Constant light activated the production of energy-related metabolites. **A** OPLS-DA analysis of metabolic shifts under different light regimes. **B** Differentially expressed metabolites in each comparison. **C** HMDB level-3 classification of the top 50 VIP metabolites in M20LL vs. M20DL. **D** Top 15 metabolites ranked by |log₂FC| in M20LL vs. M20DL. **E** HMDB level-3 classification of the top 50 VIP metabolites in M20DD vs. M20DL. **F** Top 15 metabolites ranked by |log₂FC| in M20DD vs. M20DL

To further investigate how immunity-related metabolites respond to rice blast infection under different light regimes, untargeted metabolomic profiling of rice leaves subjected to various photoperiods was performed. Principal component analysis (PCA) revealed distinct metabolic fingerprints under different light regimes (Fig. 4A), demonstrating that light conditions profoundly influence metabolite accumulation. Next, differentially expressed metabolites (DEMs) analysis revealed 84 increased and 171 decreased DEMs in the M20DL compared to the control group. The numbers were 59 and 53 increased, and 42 and 109 decreased for M20LL and M20DD groups, respectively, compared to the M20DL group (Fig. 4B). The top 50 variable importance in projection (VIP)-ranked DEMs showed that the DEMs in the M20DL group were mainly enriched in amino acids, peptides, and analogues (16%), sesquiterpenoids and diterpenoids (10%), carbohydrates and carbohydrate conjugates (10%), and fatty acids and conjugates (10%) (Fig. S3A). In addition, analysis of the top 15 most significantly altered metabolites in the M20DL group, as assessed by the |log₂FC|, found a pronounced accumulation of diterpenoids, including ent-(6α,7α,12α)−6,7,12-trihydroxy-16-kauren-19-oic acid, coetsanoic acid, and 2,3-didehydrocinnzeylanone (Fig. S3B). These results indicated that rice mounted its chemical defense primarily by accumulating diterpenoid metabolites to counteract the pathogen infection.

Similarly, compared to the M20DL group, the top 50 DEMs in the MD20LL group were mainly enriched in carbohydrates and their conjugates (12%), amino acids, peptides, and analogues (10%), indoles (8%), and purines and purine derivatives (6%) (Fig. 4C). Further analysis indicated that undecenoic acid (UA) and NMN were significantly increased after the constant light exposure (Fig. 4D). In contrast, classification of DEMs in the M20DD group revealed that 18% were carbohydrates and their analogues, 14% were amino acids, peptides, and analogues, and 6% were purines and purine derivatives (Fig. 4E). Inspection of the top 15 DEMs showed that the abundance of six carbohydrates was all decreased, with trehalose showing the largest decline, followed by trehalose-related compounds, such as 3-trehalosamine, D-mannoheptulose, and D-fructose (Fig. 4F). These results suggested that constant light may enhance rice immunity by circadian-clock-mediated elevation of NMN-related metabolites, whereas constant dark may impair immunity by reducing carbohydrate-mediated energy supply, such as trehalose.

### Constant Light Exposure Activated the NMN-Related Pathway

**Fig. 5:**
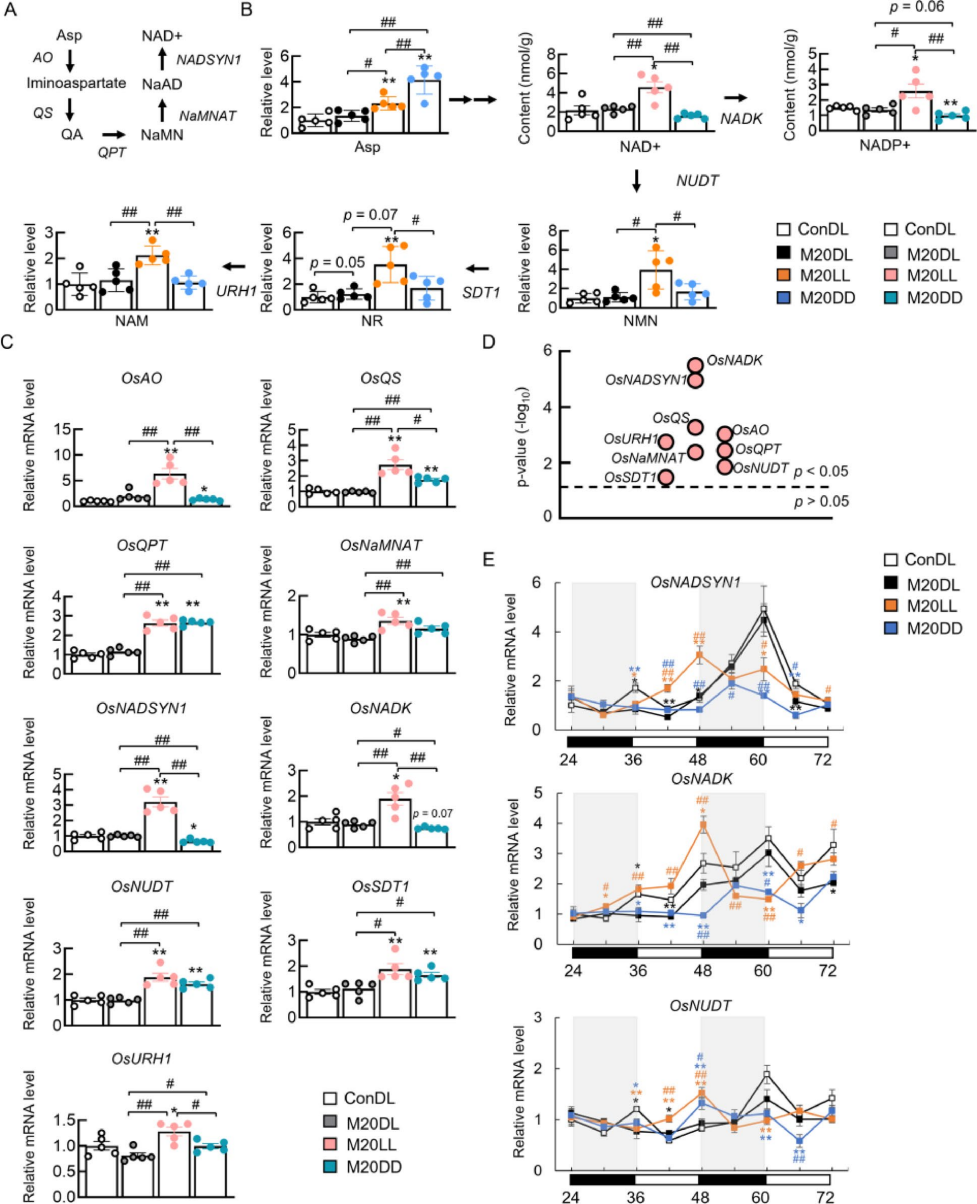
Constant light activated NMN-related pathway. **A** Metabolites and key enzymes involved in NAD⁺ metabolism. **B** Changes in NAD⁺-pathway metabolites under different light regimes. **C** Changes in NAD⁺-pathway genes under different light regimes. **D** Rhythmicity significance of NAD⁺-pathway genes, *p* < 0.05 indicates significant rhythmicity. **E** Circadian changes in core NAD⁺-pathway genes under different light regimes. Data are expressed as means ± SEM, *n* = 5 for each group for qPCR and metabolites analysis, ^*^p < 0.05, ^**^p < 0.01 vs. Con, ^#^*p* < 0.05, ^##^*p* < 0.01 vs. M20DL or as indicated

To dissect the metabolic pathway by which constant light exposure may activate the rice immunity, the NMN-related pathway was examined (Fig. 5A). Metabolite profiling revealed that both constant light and dark conditions increased the abundance of aspartic acid (Asp), the entry point of the *de novo* NAD⁺ biosynthetic pathway (Fig. 5B). In addition, constant light specifically elevated the levels of NMN, nicotinamide-β-riboside (NR), and nicotinamide (NAM) in the salvage pathway, whereas constant darkness had no significant effect on these three metabolites (Fig. 5B). Therefore, we hypothesize that constant light may activate NAD⁺ synthesis to generate NADP⁺, and that excess NAD⁺ is hydrolysed to NMN. Consistent with this hypothesis, enzymatic assays showed that constant light increased both NAD⁺ and NADP⁺ contents, while constant dark decreased them (Fig. 5B).

RT-qPCR analysis of the mRNA expression of key enzymes involved in NMN metabolism was then conducted. Constant light exposure upregulated the expression of genes in the NAD⁺ pathway, such as *Oryza sativa* Aspartate oxidase (*OsAO*), *Oryza sativa* Quinolinate synthase *(OsQS)*,* Oryza sativa* Nicotinate-nucleotide pyrophosphorylase *(OsQPT)*, *Oryza sativa* Nicotinamide mononucleotide adenylyltransferase (*OsNaMNAT*), and *Oryza sativa* NAD⁺ synthase (*OsNADSYN1*), as well as genes in the NAD⁺ salvage pathway, including *Oryza sativa* NAD⁺ diphosphatase (*OsNUDT*), *Oryza sativa* Pyrimidine- and pyridine-specific 5’-nucleotidase (*OsSDT1*), and *Oryza sativa* Uridine nucleosidase (*OsURH1*) (Fig. 5C). Constant light also induced the expression of *Oryza sativa* NAD⁺ kinase (*OsNADK*), the key enzyme for NADP⁺ synthesis (Fig. 5C). However, Constant darkness did not completely suppress the expression of these genes. Among them, *OsAO* and *OsNADSYN1* were significantly downregulated, while *OsQS*, *OsQPT*, *OsNUDT*, and *OsSDT1* were significantly upregulated (Fig. 5C).

Next, the circadian rhythmicity of the genes involved in the NMN metabolism pathway was evaluated by Metacycle. All these genes exhibited significant rhythmicity, indicating they are under circadian control (Fig. 5D). The rhythmic expression of key genes under different light regimes was further verified. Rice blast infection caused a loss of rhythmicity of *OsNADSYN1*, *OsNADK*, and *OsNUDT* under normal dark/light cycle, while constant light exposure shifted the phase of these genes and triggered an evident up-regulation from 42 to 48 h, which tended to return to regular expression after 72 h of infection (Fig. 5E). In contrast, constant dark resulted in an overall downregulation of these genes (Fig. 5E). These results suggested that constant light upregulated the circadian expression of NMN metabolism-related genes in a time-restricted way.

### NMN Alleviated Rice Blast Symptoms

**Fig. 6:**
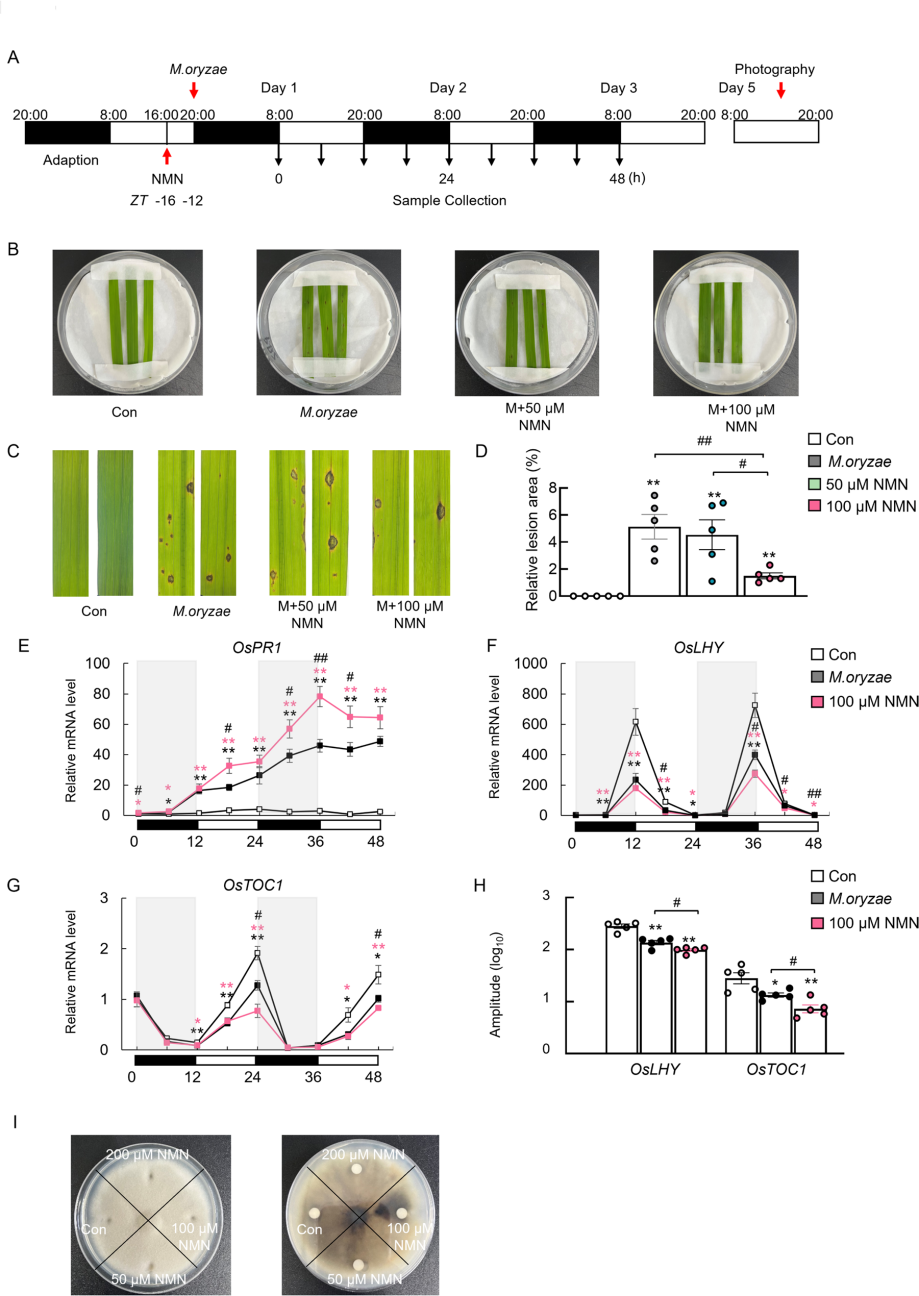
NMN alleviated rice blast symptoms and altered the rhythmic expression of circadian clock genes. **A** Experimental design. **B** Representative phenotypes of attached rice leaves 5 days after rice blast infection. **C**, **D** Leaf phenotypes after 5 days of infection and quantification of lesion areas. **E**–**G** qRT-PCR analysis of the *OsPR1* gene and circadian clock gene expression. **F** Amplitude analysis of the clock gene. **G** Plate-based antifungal assay. Data are expressed as means ± SEM, *n* = 5 for each group, ^*^p < 0.05, ^**^p < 0.01 vs. Con, ^#^*p* < 0.05, ^##^*p* < 0.01 vs. *M.oryzae*

To investigate whether constant light-induced activation of rice immunity was associated with the metabolites, NMN was selected for functional validation. Firstly, a detached-leaf assay was conducted, and 50 and 100 µM NMN were spotted onto rice leaves 4 h before rice blast infection (Fig. 6A). The results showed that both concentrations of NMN treatment alleviated blast lesions to some extent under detached conditions after 5 days of infection (Fig. 6B). Subsequently, NMN of the same concentration was sprayed on rice leaves for validation in live plants, and the leaf samples were collected every 6 h (Fig. 6A). Though 50 µM NMN failed to reduce disease symptoms, 100 µM NMN markedly attenuated rice blast lesions (Fig. 6C–D). Subsequently, the rhythmic expression of defense- and clock-related genes was examined after NMN treatment. Pretreatment with NMN significantly elevated the mRNA expression of the defense gene OsPR1 over time (Fig. 6E). Moreover, NMN further dampened the amplitude of the core circadian genes *OsLHY* and *OsTOC1*, without affecting their phase (Fig. 6F–H). Subsequently, a plate inhibition assay was conducted, which revealed that NMN at various concentrations showed no significant inhibitory effect on the mycelial growth of *M.oryzae* (Fig. 6I). Taken together, NMN might enhance rice immunity to mitigate blast symptoms without affecting fungal growth.

## Discussion

According to the literature, light irradiation can activate the hypersensitive response mediated by resistance genes, plant hormone signaling pathways, H2O2 accumulation, and the activities of ascorbate peroxidase and catalase (Chandra-Shekara et al. 2006). Specifically, under constant light (24-h photoperiod) conditions, the expression of the *PR1* gene and the SA content are higher than those under alternating light/dark cycles or constant dark in Arabidopsis (Lajeunesse et al. 2023). Consistently, our study also revealed a significant regulatory effect of light conditions on rice immune responses. Under constant light conditions, *OsPR1* expression was upregulated following *M. oryzae* infection; however, this induction effect lasted only up to 72 h, indicating that the impact of constant light on disease resistance genes is not sustained. Furthermore, this phenomenon may be closely related to the progression of *M. oryzae* infection. Within the first 72 h after inoculation, hyphae complete colonization and continue to expand (Ribot et al. 2008). Benefiting from the additional energy provided by constant light, the peak expression of *OsPR1* during the early immune response was significantly higher than that in other groups. However, after 72 h, lesion formation begins, and the defense of the plant may shift from synthesizing disease resistance proteins to mechanisms such as cell wall reinforcement and secondary metabolite accumulation, aiming to limit further damage to leaf tissues by the pathogen. A study using time-series co-expression analysis revealed 10 modules of pathogen gene expression in rice during *M. oryzae* infection from 0 to 144 h, with most genes peaking between 8 and 72 h (Yan et al. 2023). Therefore, we speculated that it is not continuous light that depletes the system; instead, the rice immune response exhibits dynamic changes tailored to the infection pattern of M. oryzae. Our previous experiments have also confirmed this point (Sun et al. 2025).

However, in the present study, H_2_O_2_ production was highest under continuous darkness, likely due to the most severe disease lesions and tissue damage under this condition. For instance, similar observations have been reported in *Capsicum annuum L*. infected with *Phytophthora capsici*, where larger lesion areas led to greater H_2_O_2_ accumulation (Yang et al. 2023). Additionally, endogenous JA content in rice showed no significant differences across different light conditions. Previous studies have shown that while plants grown under long-day conditions do not exhibit higher JA levels than those under short-day conditions, they may enhance JA sensitivity by modulating downstream JA-responsive gene signaling (Cagnola et al. 2018). Furthermore, we also found that constant light exposure initiated at 8:00 am post-infection also alleviated rice blast symptoms, suggesting that timely light administration after *M. oryzae* infection may mitigate disease severity regardless of the initial infection time. Future studies should optimize light regimes by maintaining photoperiods closer to natural conditions while ensuring unimpaired fungal infection processes to enhance translational relevance for field applications.

GO and KEGG enrichment analyses of the M20LL and M20DD groups revealed that upregulated DEGs under constant light were primarily enriched in processes such as photosynthesis and energy generation. Conversely, these processes, along with the starch and sucrose metabolism pathways, were significantly downregulated under constant dark conditions. Recent studies have demonstrated an interplay between the plant circadian clock and photosynthesis (Dodd et al. 2015; Joo et al. 2017). Photosynthesis is the process of capturing light energy and converting it into chemical energy, involving steps such as light harvesting, electron transport, carbon fixation, and ATP generation (Hwang et al. 2022). These processes rely on numerous gene products, which are typically regulated by *CCA1* (Huang et al. 2023). Importantly, the enhancement of photosynthesis and energy metabolism is closely linked to plant immunity. Multiple wheat foliar diseases reduce green leaf area, leading to decreased chlorophyll content in infected tissues (Aboukhaddour et al. 2020). This directly impairs photosynthetic capacity and the synthesis and accumulation of organic compounds in photosynthetic source organs, ultimately resulting in reduced wheat yield (Aboukhaddour et al. 2020).

Additionally, when analyzing the interaction between *Arabidopsis thaliana* and *Pseudomonas syringae*, it was found that both pathogenic and non-pathogenic bacterial strains could impair photosynthesis in *Arabidopsis thaliana* leaves. Notably, a decline in photosynthetic activity was detectable as early as 3 h post-infection (Chen et al. 2015). Chloroplast elongation factors break the growth-immunity trade-off by simultaneously promoting yield and defence (Qi et al. 2024). Meanwhile, the activation of plant immune responses is often accompanied by energy consumption and metabolic reprogramming (Deng and He 2024), which can negatively impact growth and development. Under constant light conditions, the enhanced energy supply may provide sufficient resources to support rice immunity, thereby alleviating blast symptoms. We thus propose that the circadian clock modulates rice blast resistance by coordinating photosynthesis and energy metabolism to optimize immune responses against rice blast infection.

Metabolomic analysis revealed significantly elevated NMN levels under constant light conditions. NMN is a key intermediate in the synthesis of NAD^+^, which is an indispensable coenzyme in cellular energy metabolism (Hashida and Kawai-Yamada 2019). Under illumination, the cyclic electron transport (CET) pathway rapidly establishes a trans-thylakoid proton gradient, elevating stromal pH, and then activates *NAD kinase 2* (*NADK2*), which catalyzes the phosphorylation of NAD⁺ to generate NADP⁺, thereby expanding the NADP⁺ pool in chloroplasts(Fukuda et al. 2023). Thus, we hypothesized that the sustained supply of NADP⁺ during photosynthesis activates the *de novo* biosynthesis pathway of NAD⁺ to maintain cofactor homeostasis. A growing body of evidence indicates that extracellular NAD(P)^+^ (eNAD(P)^+^) serves as a novel immune signaling molecule in plant cells, capable of activating plant immune responses and modulating systemic acquired resistance (SAR) against pathogen infection(Mou 2017). Our experimental data revealed elevated levels of both NAD^+^ and NADP^+^ under constant light conditions, accompanied by upregulation of most genes in the NAD^+^ biosynthesis pathway. Interestingly, under constnat dark conditions, not all these genes were suppressed. We speculate that the partial upregulation observed in rice under constant dark conditions may reflect the severe disease symptoms, where residual energy production remains critical for sustaining immune responses. Collectively, these findings suggested that the NAD^+^ pathway plays a positive regulatory role in rice blast defense under constant light conditions.

Furthermore, excess NAD⁺ may undergo hydrolysis to generate NMN, which also explains the observed accumulation of NMN under constant light conditions in the present study. Our experiments confirmed the activation of key genes in the NAD⁺ hydrolysis pathway, along with increased levels of NR and NMA in the metabolome. However, this study did not measure endogenous levels of NR and NMN in rice, which should be clarified in future research by developing rice-specific detection methods. Previous studies demonstrated that exogenous NMN application significantly enhances resistance against *Fusarium graminearum* in *Arabidopsis* and barley (Miwa et al. 2017). In our study, 100 µM NMN markedly alleviated rice blast symptoms and was found to feedback-regulate the circadian clock. We thus proposed that NMN likely functions as a plant immune inducer that mediates defense responses via clock modulation. However, this study has only preliminarily confirmed that NMN does not affect the growth of *M. oryzae*, while its effects on conidial production and the pathogenicity of the fungus remain unclear, which represents a limitation of this study. Furthermore, the molecular mechanisms underlying NMN-mediated alleviation of rice blast symptoms warrant further investigation.

**Fig. 7:**
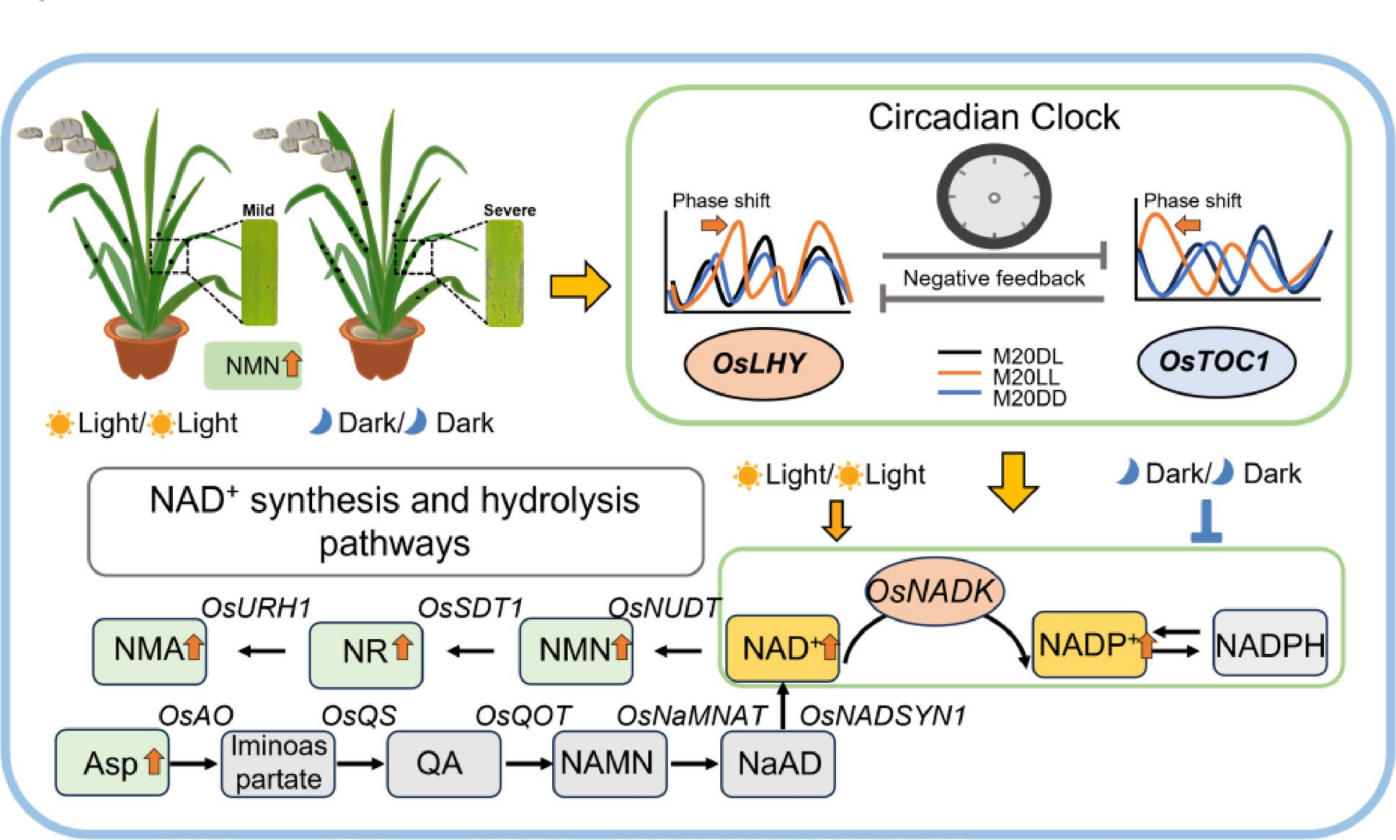
Proposed mechanism of light-mediated circadian regulation of rice immunity enhancement. Constant light exposure significantly alleviated rice blast symptoms, while constant dark exposure exacerbated disease progression. Mechanistically, persistent light remodels the circadian network by altering the phase and amplitude of core clock genes. Furthermore, constant light exposure specifically activated bidirectional regulation of NAD⁺ metabolism by promoting *de novo* NAD⁺ biosynthesis and enhancing its hydrolysis pathway. Notably, NMN, the metabolic product of this pathway, effectively alleviated rice blast severity

In summary, our study revealed that constant light exposure at different time points significantly alleviated rice blast symptoms, whereas constant dark treatment exacerbated disease progression. Mechanistically, constant light reshaped the circadian-regulatory network by altering the rhythmic characteristics of core clock genes and synchronizing the daily dynamics of photosynthesis and energy metabolism (Fig. 7). Notably, constant light specifically activated bidirectional control of NAD⁺ metabolism, simultaneously enhancing *de novo* NAD⁺ biosynthesis and its hydrolysis, with key genes in both pathways exhibiting pronounced circadian oscillations (Fig. 7). The key metabolite, NMN, was experimentally confirmed to reduce blast severity effectively. Therefore, for controlled-environment agriculture, a precisely timed lighting regimen combined with chronobiology-guided agrochemical application could optimize plant immunity and growth, offering tremendous potential to boost both crop yield and quality.

## Supplementary Information

Supplementary Material 1

## Data Availability

No datasets were generated or analysed during the current study.
