# Supplementary material for: Constant Illumination Boosts Rice Immunity via the Nicotinamide Mononucleotide Signaling Pathway: A Mechanistic Exploration
Source: Rice (N Y). 2026 Feb 20;19:20. doi: 10.1186/s12284-026-00892-x (PMC13031459; doi:10.1186/s12284-026-00892-x)
Supplement: Supplementary file 1 — Supplementary Material 1 [file 12284_2026_892_MOESM1_ESM.docx]

**Constant Illumination Boosts Rice Immunity via the Nicotinamide Mononucleotide Signaling Pathway: A Mechanistic Exploration**

Mengyan Sun^1^, Xinqing Wu^1^, Kunying Ding^1^, Lin Song^1^, Mengying Zhou^1^, Xiaoyan Su^1^, Zhi Ye^1^, Minghao Tang^1^, Tao Lu^2^, Haifeng Qian^2^, Zhengwei Fu^1^, Yinhua Ni^1,^*

^1^College of Biotechnology and Bioengineering, Zhejiang University of Technology, Hangzhou, 310032, China

^2^College of Environment, Zhejiang University of Technology, Hangzhou, 310032, China

*Corresponding author.

Yinhua Ni, PhD

College of Biotechnology and Bioengineering, Zhejiang University of Technology, No.6 District, Zhaohui, Hangzhou, Zhejiang, 310032, China

Tel: 86-571-88813845; Fax: 86-571-88813845

E-mail: shali0145@zjut.edu.cn

**Table S1.** Primer sequence for RT-qPCR

| **Geneid** | **Primer 5’ to 3’** Forward | **Primer 5’ to 3’**Reverse |
| --- | --- | --- |
| *OsLHY* | CAGATAAGGCCGACACCAAAC | GGTGTGTTGGAACCACATG |
| *OsTOC1* | TTAACTAGGCAATACATCCA | CGTCCATAGTAACAGTGAT |
| *Ubiquitin* | GCCCAAGAAGAAGATCAAGAAC | AGATAACAACGGAAGCATAAAAGTC |
| *OsPR1* | GTGTCGGAGAAGCAGTGGTA | CGAGTAGTTGCAGGTGATGAAG |
| *OsQs* | TGCGCTTCATGCCCATACAT | CTGGTAATCTCTTTGTTGCCTGG |
| *OsAO* | GCAACACGAACTACGCACAA | GCAATGAGCTCCTTCACACG |
| *OsQPT* | GGCGAAGGAAAAGCTTGCAC | CACATTTCCAGATGCCTCAGTC |
| *OsNADK* | GATGGGACTGTTTTATGGGCT | CATGGCACTGTAGACGGTTTC |
| *OsNADS* | AGATTGACTGGGAAACGCCC | CGAGCTGCAGTCATATTTCGT |
| *OsNUDT* | ATGCCATGCCAACTGATGGT | ATATGGATGGACCAGGCCAA |
| *OsSDT1* | AAGCTGCAGGCTTTCACACT | CCAACCTGCAGCAAATTCGT |
| *OsURH1* | GGGTCTCACAGTTTTCGACAC | AGCAACAGTGCTGATAGGCAT |
| *OsNaMNAT* | GTTGATCGATGGGAGGCAATG | GAATGATTCGAGCAGGTCGG |


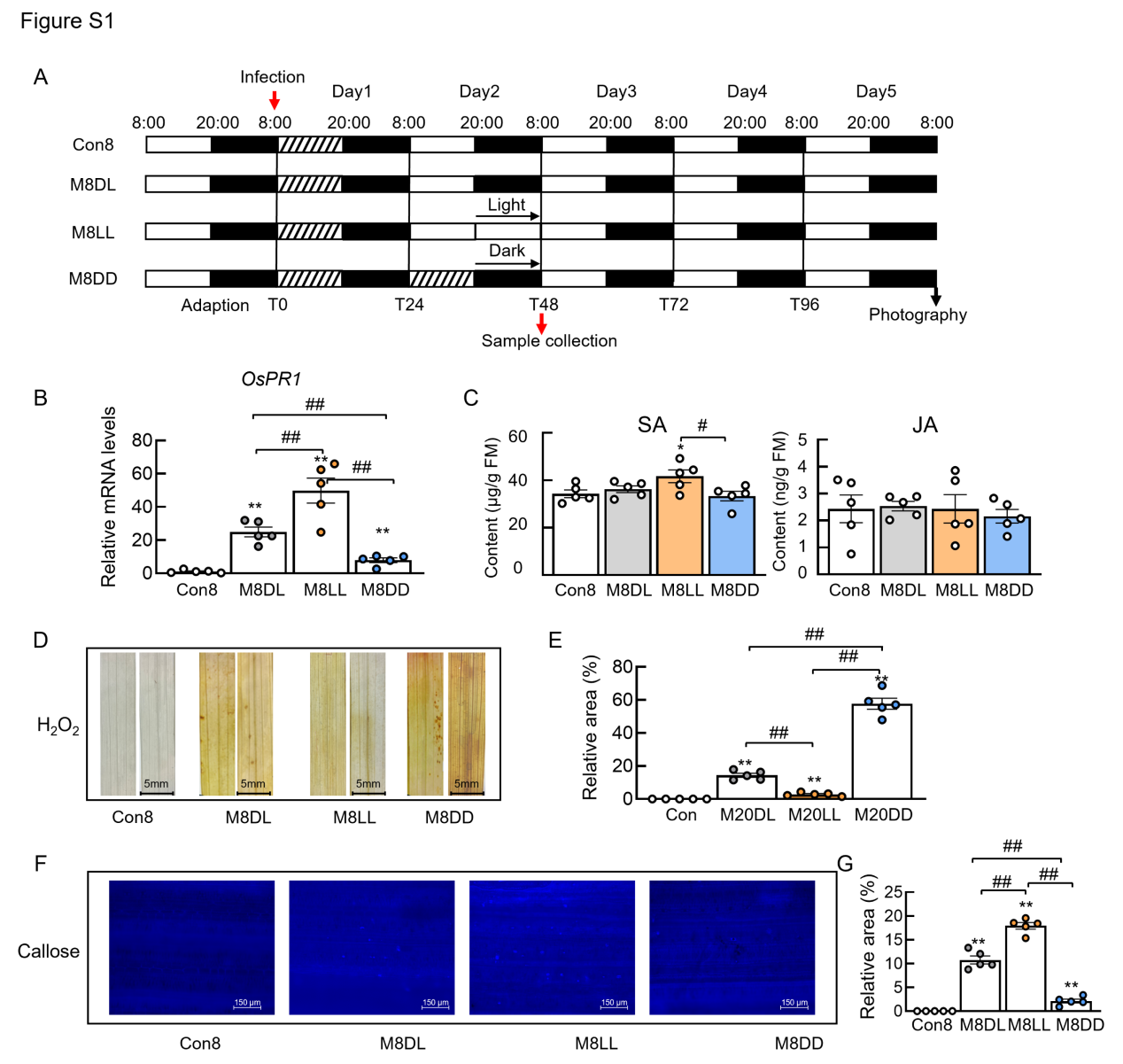


**FIGURE S1** Infection at different time points under constant light alleviated rice blast symptoms. (A) Experimental design for rice blast infection at 8:00 am, the shaded area (with slanted lines) in the diagram denotes the region of artificial darkness. (B) RT-qPCR analysis of the expression of *OsPR1* under different light regimes. (C) Quantification of the plant immune hormones SA and JA. (D) H₂O₂ accumulation, the scale bar represents 5 mm. (E) H₂O₂ quantification.（F) Callose deposition, the scale bar represents 150 μm.（G) Callose deposition quantification. Data are expressed as means ± SEM, *n* = 5 for each group for qPCR and phytohormone analysis, *n* = 5 for H₂O₂ quantification, *n* = 5 for callose deposition quantification,^*^*p* < 0.05, ^**^*p* < 0.01 vs. Con, ^#^*p* < 0.05, ^##^*p* < 0.01 vs. M20DL or as indicated.


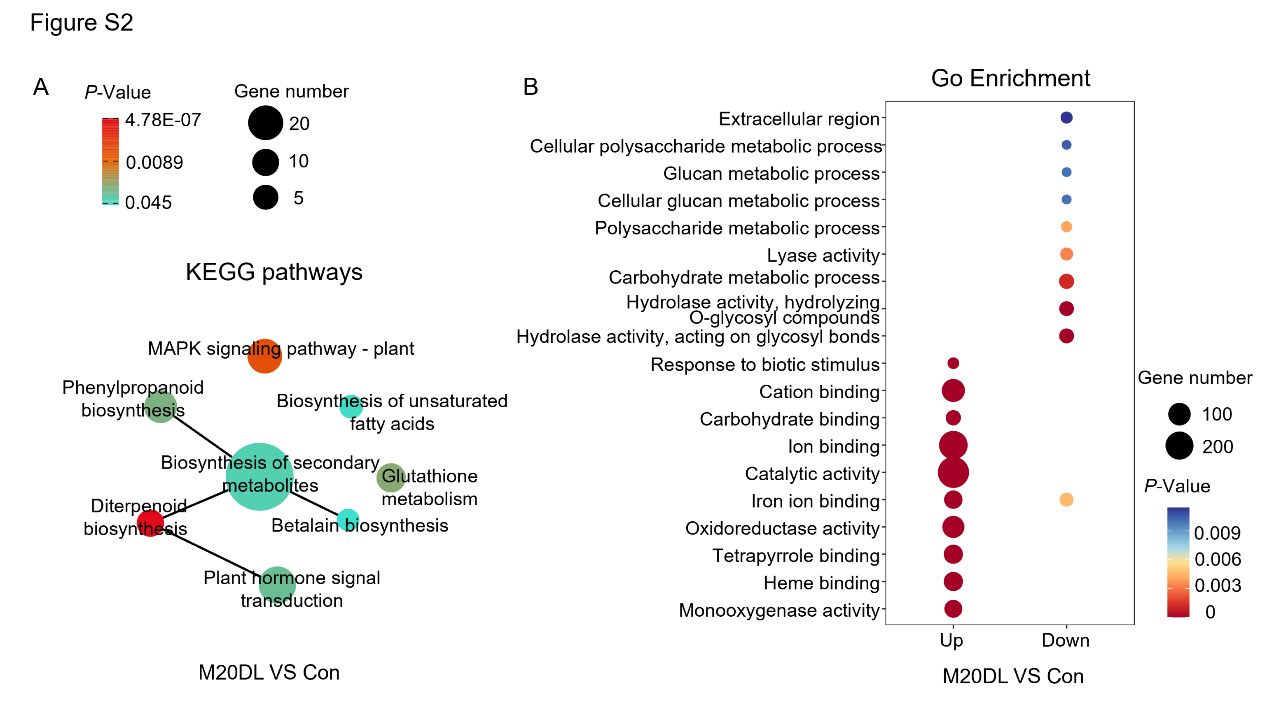


**FIGURE S2** The transcriptional patterns of rice after rice blast infection under normal light/dark cycle. (A) KEGG pathway enrichment of DEGs in M20DL vs Con. (B) GO enrichment of upregulated and down-regulated DEGs in M20DL vs Con.

**
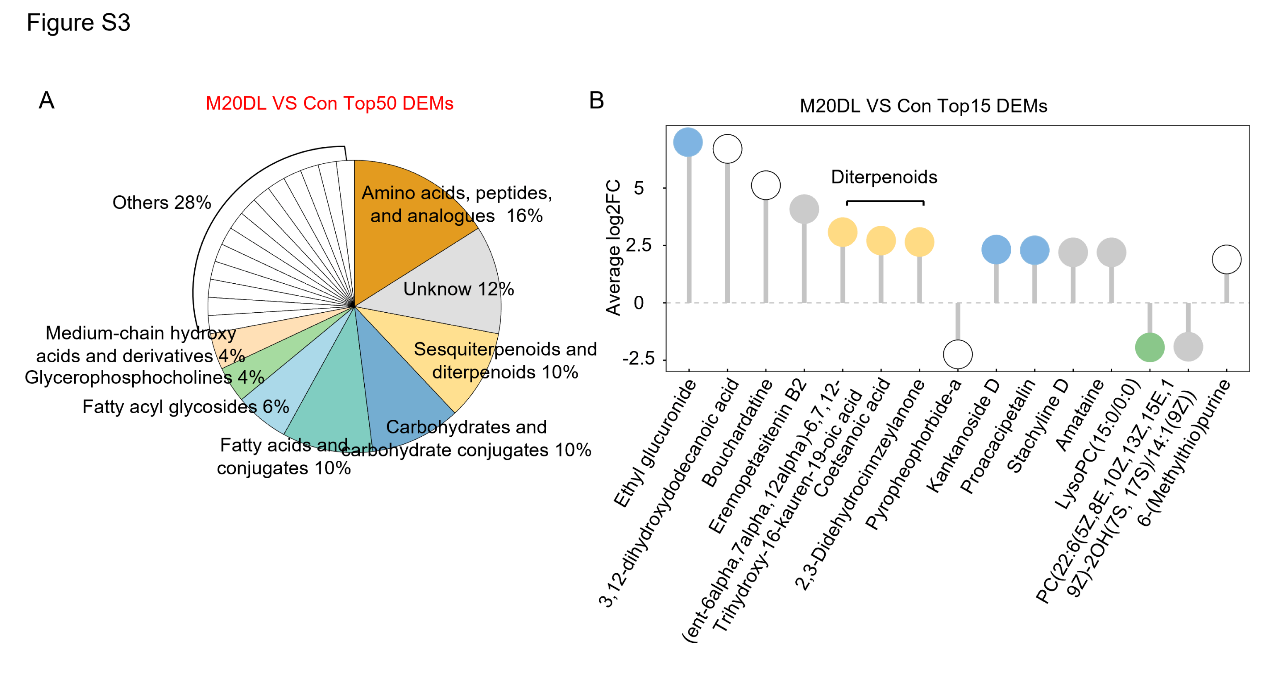
**

**FIGURE S3** Metabolomic changes in rice after rice blast infection under normal light/dark cycle. (A) HMDB level-3 classification of the top 50 VIP metabolites in M20DL vs Con. (B) Top 15 metabolites ranked by |log₂FC| in M20DL vs Con.
